# Cursory Remarks on a Bill Now in the House of Peers for Regulating of Mad Houses, with Observations on the Defects of the Present System

**Published:** 1817-10

**Authors:** 


*Cursory Remarks on a Bill now in the House of Peers for Regulating of Mad Houses, with Observations on the Defects of the present System.* By GEORGE MAN BURROWS, M. D. F. L. S. &c. *Octavo, pp.* 104. London, 1817.

PERHAPS there is no subject connected with our internal police that more deserves the attention of the Legislature, than the one which this Bill professes to embrace. In its enactments all are interested, and its provisions come home to the bosoms of every class, and almost of every individual in the community, seeing the visitations of insanity are indiscriminate and unsparing, and its victims, of whatever rank, are reduced to one common level of pitiable, and very often hopeless, degradation. We therefore cannot but honour that benevolent and paternal spirit, in which Parliament instituted an inquiry into the state of Mad Houses throughout the kingdom, as emanating from a sincere desire to satisfy the wishes of the most respectable part of the community, and to improve the moral and physical condition of the insane. It cannot be doubted for a moment, that their intention was good : but, in matters political, rectitude of intention is not enough ; for an anxious or too intermeddling spirit, on the part of the Legislature, now and then tends to produce greater inconveniences than those which, from the best of motives, were desired to be removed.

With some such doubtful impression we have always regarded the Bill in question ; and though by no means blind to the abuses, or callous to the enormities, which have been ascertained to exist in *some* of the establishments for the care and management of the unhappy subjects of mental derangement, yet we have never suffered ourselves to be hurried by popular clamour into that degree of *desperation,* which would *explode* the present system before it had laid the foundation of a better ; being convinced that it is, in general, much more easy to point out what is wrong, than to substitute what is right ; and to broach hypothetical schemes of utility, than to establish what is at once useful and practicable.

We, therefore, quite agree in opinion with Dr. Burrows with regard to this proposed Bill. He has gone at great length into the subject in the pamphlet before us, and has pointed out, in the strongest manner, the clumsiness and bad policy of the Act now in contemplation. But, besides, he has given to his little work a degree of interest much greater than the transient sort which its title might lead some to attach to it; having interspersed in it many remarks which may tend to introduce a more improved method of treating insanity, and, in the mean time, must be extremely useful to those who have the arduous charge of patients in that lamentable state. Furthermore, we are disposed to think highly of that tone of *right feeling* which Dr. B. displays throughout his performance; it is greatly creditable to him, and evinces at once the soundness of his understanding and the integrity of his motives; and must add much weight to his opinion on a subject, about which (if we may judge from the sensation existing throughout the country) it is difficult even for the wisest to be cool, or the best to be dispassionate.

That our Readers may be able to estimate the force of Dr. B's objections to the Bill in question, we shall lay before them some of its principal clauses.

"In order that proper persons may be appointed for licensing such Houses as may be kept for the reception of Lunatics, other than Lunatic Asylums before mentioned, and visiting such houses and all public Hospitals, within that part of the united kingdom called *England;* Be it enacted, that his Majesty's principal secretary of state for the home department shall annually, on the last day of September of every year, or within ten days then next following, appoint eight persons to be Commissioners for that part of the united kingdom; of whom, four at the least [—*Here we would ask, why not the whole eight?*—] shall be Fellows or Licentiates of the College of Physicians in London or Edinburgh, as Commissioners for granting licenses within the said part of the united kingdom, and for visiting the several houses therein for the reception of insane persons within the same, and all public hospitals for the reception of such persons, in such districts or divisions in England as shall be allotted by his Majesty's principal secretary of state, so that there shall be two of such Commissioners appointed for each district, one of whom shall be a Physician; and shall also appoint a person to act as secretary to the said commissioners at their general meetings: And the said eight commissioners so to be appointed, shall be, and are hereby declared to be, commissioners for granting licenses within the said part of the united kingdom for the year then next ensuing; provided that two at least of the commissioners to be so appointed, shall be persons who have not acted as commissioners for the preceding year; and that no person whatever shall be capable of acting as a commissioner for more than four years, unless he shall be specially authorized for that service by a new appointment from the principal secretary of state." Bill, p. 2.

Another section follows, empowering the secretary of state to appoint six additional commissioners, for the purpose of visiting *only*, all public hospitals and licensed asylums for the reception of the insane; but from such appointment these commissioners are to receive "no emolument whatever."

Now, to all this Dr. B. objects that the districts will be too extensive, and occupy too much of the commissioners' time in traversing and inspecting, to the great detriment of their professional avocations and other pursuits. Besides, if their visitations are not made oftener than once a year, the office will be nugatory: on the other hand, if they are performed with due frequency, the expence will be enormous. In the present state of our profession, who could be found willing to execute such an office? A young physician, were he even in every respect fit for it, would infallibly regard it as a bar to his professional career, as a mill-stone about his neck, which must keep him down from that station among his compeers, which ability and undivided attention may hope, sooner or later, to attain. Again, physicians in the meridian of life are generally better and more lucratively engaged; and few of them, we think, would be so silly as to barter the steady income they derive from their profession, for the *intermitting* salary and barren honour of a commissionership! A physician's time is, in fact, his property, and it is reasonable to suppose that he will put a corresponding value upon it. It is, unquestionably, a hardship to impose duties which must occupy that time, without an adequate remuneration. Upon the whole then, we think that the duty of *licensing*, a medical man might perform without such very great reluctance; but the office of a *visiting* commissioner would be one of a much more unpalateable nature; inasmuch as, according to the proposed plan, it would go to destroy that pleasing connection between *visit* and *fee*, (a connection hitherto subsisting in right comfortable reciprocity,) which we should be sorry to see ever *interrupted*, far less, totally *dissolved!* On the contrary, "*esto perpetua*" is the sincere wish of our hearts on this very interesting and really *vital* point!

In addition to the eight regular commissioners, and the six supernumerary ones, the Bill, (at p. 4) enacts, that two of the justices of the peace, acting for the county or place where such licensed establishments shall happen to be, shall have the power of visiting and inspecting these establishments at their pleasure, without any regard to time or form.

"And such justices, so visiting as aforesaid, shall have at all times, and may use and exercise, such powers and authorities in visiting and examining any such houses and hospitals, and the premises thereto belonging, and the *persons confined therein;* and also in examining upon oath the keeper or keepers of such houses and hospitals, and the attendants therein, as are by this act given to the commissioners under this act."

Again, it is enacted, that any keeper or superintendant of a mad house who shall be refused a license, or who may have been judged to have forfeited his license, and thinks himself aggrieved, may appeal to the next general quarter sessions of the county or division in which the house shall be situated, and he is to give sufficient securities to abide the the orders and award of the said court; and the said justices "shall, in a summary way," finally hear and determine the said appeal; but no proceeding "shall be quashed or vacated for want of form, or be removed by *certiorari*, or by any other writ or process whatsoever, into any of his Majesty's courts of record at Westminster, or in Edinburgh, or elsewhere."

To these clauses Dr. Burrows has objected at great length, and in terms at once spirited and sensible. But indeed, very little argumentation is required to expose the great faultiness of such provisions, as they carry along with them *prima facie* evidence of hastiness and impolicy. We have only room barely to hint at their defects.

1. They open a very wide door for partiality and private oppression on the part of magistrates and visiting commissioners; and although the station and character of such gentlemen may be thought a guarantee for the right execution of the trust reposed in them, still it is repugnant to the immutable principles of justice, and to the spirit of our constitution, that any man, in the responsible situation of a superintendant of an establishment of this sort should, in his property and character, be at the mercy of any individual, however virtuous or well intentioned.

2. These frequent visitations of inquisitorial scrutiny, must harass the insane, and keep up in their minds a spirit of activity, if not of irritation, highly unfavourable to their recovery.

3. It may, and in many cases will, make the superintendant the victim of his malicious, perhaps profligate servants; who will glut their private revenge by giving evidence that must ruin his character and property. As for that part of the clause which says that the evidence of the *lunatics themselves* shall be received as valid against their keeper, it is so "passing strange," that we cannot cease to wonder how any thing so weak could find its way into an act of parliament.

4. The act gives power to the plaintiff to seek justice in any form or in any court that he chooses, against the superintendant, but it denies the latter the same advantage. Hence it is evident (as our author very properly remarks,)

"that the proprietor or superintendant of any licensed house, besides conforming to all the regulations visitations, expence, pains, penalties, forfeitures, losses, vexations and traductions, to which the former part of this act will expose him, is also to be liable to all the litigious, and even criminal proceedings, to which he was before subjected by virtue of any pre-existing act of parliament." p. 17.

5. The result of all this will be, that instead of the condition of the insane being benefited by the proposed change, it will probably be quite the reverse; for we can foresee, that they will, too often, fall into the hands of ignorant, low, self-interested persons, quite incapable of conducting either the medical or moral means of curing their disease. What respectable man, particularly if he is of our profession, would stoop to embark his character and property, as a superintendant of an establishment of this kind, when he considers the perilousness of the speculation, and the thousand mortifications, not to say penalties, to which he must be constantly exposed? A man that would meanly submit to such, is not fit for the office; and he who has a mind adequate to the charge, would not subject himself to that species of domiciliary tyranny which this act proposes to establish into a law. Sycophancy and submissiveness are not the leading qualifications of a superintendant.

"He ought, (as Dr. B. judiciously remarks) to possess an excellent understanding; the more cultivated the better; tempered manners; vigilance; courage; and great presence of mind. He should have endowments of nature, not mere acquirements; he should entertain a full impression of the divine precepts of pure religion; be practically moral himself; and enforcing it by his example upon those under him. Such a man indeed is rare! But if he be found, will he become a superintendant, and subject himself to the proposed law? Decidedly, he will not." p. 55.

We are the more willing to give Dr. B's idea of what a superintendant ought to be, in his own words, because he considers that the great requisite to reformation in mad houses, consists in the selection of persons properly qualified for this important trust. All other regulations must be very secondary and unimportant, compared with this primary and essential one. On this head, however, we have much reason to complain of the doctor's brevity. We confess we should have liked to have heard his opinion at greater length, with regard to those improvements which he would suggest for the melioration of the insane. At p. 96, he tells us, that after five and twenty years extensive practice, it was his wish to have formed a private establishment for lunatics, which should have had every requisite. His plan therefore must be matured; and such being the case, we can see no reason for his withholding it from the public. Nay, we would submit it to his consideration, whether he is not, in some measure, bound to make known his own improved plan, after having criticised the one proposed in this act, with so much freedom, and, as we think, with so much justice.

The following observations are so excellent in themselves, and bear so forcibly on the commonly received unphilosophical notions about the pathology of insanity, that we cannot but insert them at length, although we have already exceeded our limits.

"May not medical practice have bowed too lowly at the shrine of philosophy throughout the whole of the seventeenth and part of the eighteenth centuries; the philosophers and physicians who flourished, and eminently adorned the age, were deeply involved in speculations upon the seat of the soul, and with metaphysical disquisitions upon its materiality, or the relation of mind with matter. This famous dispute ended by the triumph of *Descartes* over the advocates of materialism, and the establishing of the independency of mind upon matter. Hence the mind has been treated as a substance possessing distinct properties, and subject to the infirmities of disease. The opinion of *Descartes* and his followers became a favourite doctrine; it was taught in the schools, adopted as a dogma of alma-mater, and it has continued to pervade both precept and practice to this epoch.

"When physicians became entangled within the vortices of metaphysics, every thing was explained agreeably to the new philosophy. Hence, the aberrations of the intellect were arranged as specific diseases, and the research for first causes was overlooked in the contemplation of their effects. The treatment of insanity consequently retrograded; for what are remedies if prescribed to symptoms only, and the causes remain concealed or neglected.

" Nothing is more distant from my intention, than to enter into the mazes of metaphysics: but I shall venture an opinion, that the true obstacle to the establishing of a correct theory of the causes, and of a sound practice in the treatment of insanity, is the impression, that the mind can become diseased, independent of the body. There is not a single proof that will substantiate such a position. Insanity always originates in a corporeal cause; derangement of the intellectual faculties is but the effect. Disease of the mind, therefore, as a primary affection, is a chimera existing only in the brains of poets, pseudo-philosophers, and metaphysicians.

" To those who have before professed similar opinions, I am aware materialism has been objected: to this I have to answer only, that it is the objection always urged when nothing else can be advanced to get rid of an unanswerable argument. I am not regardless of the opinion of the world upon this point; and trust my principles are sufficiently known to exempt me from suspicion. p. 101.

After such superior sense as the above passage contains, we certainly were not prepared for its concluding sentence, and are greatly mortified that a man like Dr. Burrows should have written any such. We would here ask him, in his own words, if he has not, in this instance, " bowed too lowly" to *la haute et la basse canaille,* and mistaken their opinion for that of the world at large. We are always sorry when we see men of science descend from their native high ground, and stoop to any thing like a *peace-offering* to the dæmon of deep-rooted error. It is what, we trust, *we* shall never do; " our voice is still for war," open war, until error, however consecrated by antiquity, shall hide its head.

We always love to speak out frankly, and can assure Dr. B. if it will be any pleasure to him, that we are entirely of his opinion on this matter; and that we most potently believe, that the primary disease, or morbid cause, in every instance of mania, exists in the *brain,* and that the derangement of the *mental* faculties is merely an effect, symptom or result. If any one shall impeach the soundness of our principles for this, we shall, in return, go nigh to impeach the soundness of his understanding! Should any sciolist, piquing himself on his fancied metaphysical acuteness, or superiority as a reasoner, accuse us of confounding mind and matter, we can only answer (were it worth while to answer at all!) that we do not identify them. We are convinced they are perfectly different. We never did, never will, maintain the absurd proposition that " matter peculiarly arranged, may think." But we hold, that, *in this present state*, the immaterial and immortal mind never can operate except through the medium or instrumentality of matter. This is a law of nature; simple, primary, and without exception; and we should as soon expect to see the principle of *life*, in its separate essence, unconnected with the organization which it animates, as to see *mind* displayed, independently of that portion of the nervous system assigned by the author of our being, for the *medium* of its operations.

Besides, does not the very idea of *mind* and of *immateriality* imply a perfect exemption from disease or decay of any kind? We would like to hear how the supporters of the vulgar doctrine of " mental complaints" will reconcile their opinions with even their own notions of sound belief: for if the mind can become diseased, as they maintain, it follows inevitably, that the mind can die! Since this conclusion results from their doctrine, we may fairly be permitted to ask, *who are materialists now?*

That the mind shall, at one and the same time, be immaterial, and subject to diseases, is to us a contradiction in terms; and for such metaphysical absurdities, we, for our parts, shall always fearlessly use the caustic, or the knife, leaving it to others, if they will, and needs must, to apply to them *lenient* censures, so timid and unctuous, as almost to be mistaken for compliance or compliment. On this important subject, we have uniformly held the same opinion; of which our readers may convince themselves, by referring back to the first volume of our Journal, p. 119, (for February, 1816.)

Upon the whole, we have found in this pamphlet much to approve, with little or nothing of a contrary nature. Perhaps a more luminous arrangement might have been adopted; but as it is, we can safely recommend it to our readers. Dr. B. records in a note (at p. 77) a circumstance with regard to the medical management, or rather *mis*management of the parish poor, which, if *true*, in our opinion as well deserves the notice of the legislature, as any enormity that ever was practiced within the walls of a mad house. Such aggravated inhumanity sets all terms of reprobation at defiance!

